# Parietal white matter lesions in Alzheimer’s disease are associated with cortical neurodegenerative pathology, but not with small vessel disease

**DOI:** 10.1007/s00401-017-1738-2

**Published:** 2017-06-21

**Authors:** Kirsty E. McAleese, Lauren Walker, Sophie Graham, Elisa L. J. Moya, Mary Johnson, Daniel Erskine, Sean J. Colloby, Madhurima Dey, Carmen Martin-Ruiz, John-Paul Taylor, Alan J. Thomas, Ian G. McKeith, Charles De Carli, Johannes Attems

**Affiliations:** 1 Institute of Neuroscience, Campus for Ageing and Vitality, Newcastle University, Newcastle upon Tyne, NE4 5PL UK; 2 Department of Biology, University of Granada, Granada, Spain; 3 School of Biology, University of St Andrews, Fife, UK; 4 Department of Neurology, University of California, Davis, CA USA

**Keywords:** White matter lesion, White matter hyperintensity, Alzheimer’s disease, Small vessel disease, Hyperphosphorylated tau, Wallerian degeneration

## Abstract

Cerebral white matter lesions (WML) encompass axonal loss and demyelination, and the pathogenesis is assumed to be small vessel disease (SVD)-related ischemia. However, WML may also result from the activation of Wallerian degeneration as a consequence of cortical Alzheimer’s disease (AD) pathology, i.e. hyperphosphorylated tau (HPτ) and amyloid-beta (Aβ) deposition. WML seen in AD have a posterior predominance compared to non-demented individuals but it is unclear whether the pathological and molecular signatures of WML differ between these two groups. We investigated differences in the composition and aetiology of parietal WML from AD and non-demented controls. Parietal WML tissue from 55 human post-mortem brains (AD, *n* = 27; non-demented controls, *n* = 28) were quantitatively assessed for axonal loss and demyelination, as well as for cortical HPτ and Aβ burden and SVD. Biochemical assessment included Wallerian degeneration protease calpain and the myelin-associated glycoprotein (MAG) to proteolipid protein (PLP) ratio (MAG:PLP) as a measure of hypoperfusion. WML severity was associated with both axonal loss and demyelination in AD, but only with demyelination in controls. Calpain was significantly increased in WML tissue in AD, whereas MAG:PLP was significantly reduced in controls. Calpain levels were associated with increasing amounts of cortical AD-pathology but not SVD. We conclude that parietal WML seen in AD differ in their pathological composition and aetiology compared to WML seen in aged controls: WML seen in AD may be associated with Wallerian degeneration that is triggered by cortical AD-pathology, whereas WML in aged controls are due to ischaemia. Hence, parietal WML as seen on MRI should not invariably be interpreted as a surrogate biomarker for SVD as they may be indicative of cortical AD-pathology, and therefore, AD should also be considered as the main underlying cause for cognitive impairment in cases with parietal WML.

**Electronic supplementary material:**

The online version of this article (doi:10.1007/s00401-017-1738-2) contains supplementary material, which is available to authorized users.

## Introduction

Cerebral white matter lesions (WML) are a frequent finding at post-mortem histological examination of brains from both demented and non-demented elderly. They primarily encompass loss of integrity of the cerebral white matter due to rarefaction, which morphologically appear as demyelination when visualized with  histochemical myelin stains and may be due to loss of the axonal myelin sheath or axonal loss [25]. WML appear as white matter hyperintensities (WMH) on pre- and post-mortem T2-weighted magnetic resonance imaging (MRI) [27], and as altered diffusivity on diffusion tensor imaging (DTI) [7]. The burden of WMH increases with age [50] and is associated with cognitive decline and risk of dementia [14]. In Alzheimer’s disease (AD) patients WMH are found in up to 89% of cases [5] and are more severe than WMH seen in the non-demented elderly [40, 67].

WML/WMH are usually assumed to represent ischemia-associated demyelination and axonal loss due to arteriopathy of the white matter arteries and arterioles, i.e. small vessel disease (SVD) [47]. Therefore, the detection of in vivo WMH often supports the diagnosis of cerebrovascular disease and, in patients with cognitive impairment, the diagnosis of vascular cognitive impairment/dementia (VCI/VaD) over other forms of dementia such as AD [41]. However, previous neuroimaging [34, 51] and neuropathological studies [40] suggest a multifactorial aetiology of WMH, in addition to SVD it may also include degenerative axonal loss secondary to the deposition of cortical AD pathology, i.e. hyperphosphorylated tau (HPτ) and amyloid-beta (Aβ). Here, axonal loss in white matter could either occur as a consequence of cortical neuronal loss (later stages of AD), or as a result of retrograde Wallerian degeneration, i.e. rapid irreversible fragmentation and destruction of the distal axon [38]. One of the main triggers of Wallerian degeneration in AD is the disruption and/or blockage of anterograde fast axonal transport [55]. Eventually impaired axonal transport leads to axonal dysregulation and increased intracellular calcium concentration activating the calcium-dependent cysteine protease calpain that mediates cytoskeletal breakdown, demyelination and axonal fragmentation [12, 13, 39].

In-vivo DTI studies support the concept of AD-associated axonal changes as they have demonstrated specific topographical white matter changes, i.e. reduced axonal integrity, axonal swelling and demyelination that occur exclusively in AD patients. These changes include reduced integrity in white matter tracts connected to the medial temporal lobe (MTL) [2, 31], the cingulum and in the tracts of the temporo-parietal regions such as the superior longitudinal fasciculus [1, 7]. These changes in white matter structure have been directly associated with Wallerian degeneration independent of cortical atrophy [3, 7, 28]. Moreover, AD cases with severe cortical AD pathology and severe WML exhibited only minimal SVD pathology at post-mortem examination [17, 40], indicating that SVD was not the primary cause of WML in these cases.

In addition, cerebral amyloid angiopathy (CAA), a sub-type of SVD characterized by the accumulation of Aβ in leptomeningeal, cortical and capillary vessel walls is seen in 80–100% of AD cases [4] was suggested to cause ischemia of the white matter as the presence of CAA has been shown to be associated with increased demyelination [59] and AD patients with CAA had higher burdens of WMH compared to AD patients without CAA [26]. However, others found no relationship between WMH and CAA [56] and the relationship between CAA and the pathogenesis of WML has not yet been clarified.

The underlying aetiology of WML is not restricted to SVD and may indeed be indicative of AD pathology. However, it is not clear whether WML associated with cortical AD pathology differ in their pathological and molecular signatures from WML that are associated with SVD and such data are needed to further our understanding of the pathogenesis of WML. This is crucial as the current clinical criteria for VaD/VCI [22] and mixed AD/VaD [16] are based on the detection of WMH, which are invariably interpreted as a surrogate marker of SVD resulting in misdiagnosis with a detrimental impact on the choice of therapeutic interventions. Therefore, we investigated post-mortem brain tissue of AD and aged controls to identify differences in the composition and aetiology of WML in the parietal lobe using pathological and biochemical methods.

## Methods

### Study cohort

Our study cohort consisted of 55 human post-mortem brains (mean age 84.02 ± 7.94 years; male: 24, female: 31). During life, all dementia subjects underwent clinical assessments by board certified Old Age Psychiatrists or Neurologists and most were assessed in prospective research studies with repeated cognitive evaluation including the Mental State Examination (MMSE) [19] (MMSE scores were available for 18 out of 27 AD cases and for 6 out of 28 controls). All cases had a clinical review of records after death (AJT) at Newcastle Brain Tissue Resource (NBTR) and control subjects showed no evidence of cognitive impairment and had normal everyday functioning up until death. AD cases were confirmed as having a clinical dementia due to AD. Brain tissue was obtained at autopsy and stored within the NBTR in accordance with Newcastle University Ethics Board (The Joint Ethics Committee of Newcastle and North Tyneside Health Authority, reference: 08/H0906/136). After autopsy the left hemisphere, brainstem and cerebellum were dissected in coronal planes approximately 0.7 cm intervals and snap frozen between copper plates at −120 °C and stored at −80 °C. The right hemisphere, brainstem and cerebellum were immersion fixed in 10% buffered aqueous formaldehyde solution for 6 weeks and then subsequently dissected in coronal planes approximately 0.7 cm intervals and paraffin-embedded. All brains underwent neuropathological assessment according to the National Institute on Aging-Alzheimer’s Association (NIA-AA) criteria [44] that included assessment of Thal phases of Aβ deposition [57], Braak staging of neurofibrillary pathology [10] and Consortium to Establish a Registry for Alzheimer’s Disease (CERAD) scoring [43]. Additional neuropathological scoring of Lewy body pathology [9, 42], vascular pathology contribution to cognitive impairment (vascular impairment neuropathological guidelines (VCING)) [53], TDP-43 inclusions [30] and CAA [46] was performed. Cases did not contain any infarcts or cerebral haemorrhages. The final clinico-pathological diagnosis was AD in 27 cases, while no cognitive impairment and only mild age-associated neuropathological changes were seen in 28 cases (controls) (Table 1).

**Table 1:** Demographic and neuropathological characteristics of study cohort

	AD	Control	Statistic_*(df)*_, *p* value
Cohort number	27	28	
Mean age, years (±SD)	84.01 (6.84)	84.04 (9.00)	*t*_(53)_ = 0.17, *p* = 0.987
Gender M:F	11:16	13:15	*χ*_(1)_^2^ = 0.181, *p* = 0.671
Mean PMD, hours (±SD)	51.53 (23.70)	53.00 (24.84)	*t*_(31)_ = 1.73, *p* = 0.864
Thal Aβ phase [57]	Phase 4, *n* = 2 Phase 5, *n* = 25	Phase 0, *n* = 8 Phase 1, *n* = 9 Phase 2, *n* = 5 Phase 3, *n* = 3 Phase 4, *n* = 2 Phase 5, *n* = 1	*U*_(53)_ = 16.5, *p* = 0.0001
Braak NFT stage [10]	NFT stage 5, *n* = 3 NFT stage 6, *n* = 24	NFT stage 0, *n* = 2 NFT stage 1, *n* = 3 NFT stage 2, *n* = 11 NFT stage 3, *n* = 11 NFT stage 4, *n* = 1	*U*_(53)_ = 0.500, *p* = 0.0001
CERAD [43]	C, *n* = 27	Negative, *n* = 23 A, *n* = 3 B, *n* = 2	–
NIA-AA [44]	High, *n* = 27	No, *n* = 8 Low, *n* = 18 Intermediate, *n* = 2	–
Braak LB stage [9]	LFB stage 0, *n* = 25 LFB stage 1, *n* = 0 LFB stage 2, *n* = 1 LFB stage 3, *n* = 1	LFB stage 0, *n* = 24 LFB stage 1, *n* = 1 LFB stage 2, *n* = 1 LFB stage 3, *n* = 2	–
McKeith criteria [42]	No LBD, *n* = 25 Amygdala predominant, *n* = 2	No LBD, *n* = 24 Brainstem, *n* = 2 Amygdala predominant, *n* = 2	–
VCING criteria [53]	Low, *n* = 28	Low, *n* = 25 Moderate, *n* = 2	–
TDP-43 in AD score [30]	Stage 0, *n* = 12 Stage 1, *n* = 3 Stage 2, *n* = 4 Stage 3, *n* = 2 Stage 4, *n* = 6	Stage 0, *n* = 21 Stage 1, *n* = 3 Stage 2, *n* = 1 Stage 3, *n* = 1 Stage 4, *n* = 2	–
CAA score [46]	Stage 0, *n* = 1 Stage 1, *n* = 3 Stage 2, *n* = 12 Stage 3, *n* = 9 Stage 4, *n* = 3 CapCAA absent, *n* = 1 CapCAA present, *n* = 26	Stage 0, *n* = 15 Stage 1, *n* = 5 Stage 2, *n* = 8 CapCAA absent, *n* = 15 CapCAA present, *n* = 13	–
MMSE (±SD) [19]	5.82 (5.36)	26 (4.34)	*t*_(21)_ = 8.269, *p* = 0.0001

*AD* Alzheimer’s disease, *df* degrees of freedom; *t* Independent samples test, *X* ^*2*^ Chi -squared test, *F* female, *M* Male, *U* Mann–Whitney *U* test, *PMD* post mortem delay, *Aβ* amyloid-beta, *NFT* neurofibrillary tangle, *CERAD* Consortium to Establish a Registry for Alzheimer’s Disease, *NIA-AA* National Institute on Ageing-Alzheimer’s Association criteria for AD neuropathologic change, *LB* Lewy body, *VCING* vascular cognitive impairment neuropathological guidelines, *CAA* cerebral amyloid angiopathy, *CapCAA* capillary CAA, *MMSE* mini mental state examination

### Histological procedures

Paraffin-embedded serial sections from parietal blocks (Brodmann area 40/22) were cut at 6 μm thickness and mounted onto superfrost plus charged glass slides (Thermo Shandon, Cheshire, UK). The parietal lobe was selected as AD patients frequently show WMH in the posterior region inclusive of the parietal white matter [67] and this region contains large amounts of white matter for pathological assessment. Sections underwent histological staining with Bielschowsky silver stain (cold method) [37] for assessment of axonal density, myelin stain luxol fast blue (LFB) to assess WML area and myelin pallor (as a measure of demyelination) and haematoxylin and eosin (H&E), for visualisation of white matter artery/arteriole walls for assessment of SVD. Immunohistochemistry was performed for chronic axonal transport dysfunctional marker non-phosphorylated SMI32 (antibody SMI32) [36, 63], as well as for HPτ (antibody AT8) and Aβ peptide (antibody 4G8) (details on primary antibodies and antigen retrieval protocols for immunohistochemistry are presented in Table 2). Immunopositivity was detected using the Menarini X-Cell-Plus HRP Detection Kit (Menarini Diagnostics, Winnersh-Wokingham, UK) with 3,3 diaminobenzidine (DAB) as a chromagen and haematoxylin as a counter stain. All histologically and immunohistochemically stained sections were subsequently dehydrated through a series of alcohols, cleared and mounted using DPX (CellPath, Powys, UK).

**Table 2:** Details of primary antibodies and protocols

Primary antibody	Source	Target	Species	Primary antibody dilution	Antigen retrieval protocol
Anti-neurofilament H non-phosphorylated SMI32	Millipore, CA, USA	180-200 kDa non-phosphorylated neurofilament H protein	Mouse monoclonal	1:1000	De-parraffinised sections microwaved in tris-buffered saline (pH 9.0) for 15 min
AT8	Innogenetics, Ghent, Belgium	Phospho-PHF-tau pSer202 + Thr205	Mouse monoclonal	1:4000	De-parraffinised sections microwaved in 0.01 mL citrate buffer for 10 min
4G8	Signet Labs, Dedham, MA, USA	Amyloid 17-24	Mouse monoclonal	1:15,000	De-parraffinised sections were immersed in concentrated Formic acid for 1 h

### Image analysis

**Fig. 1:**
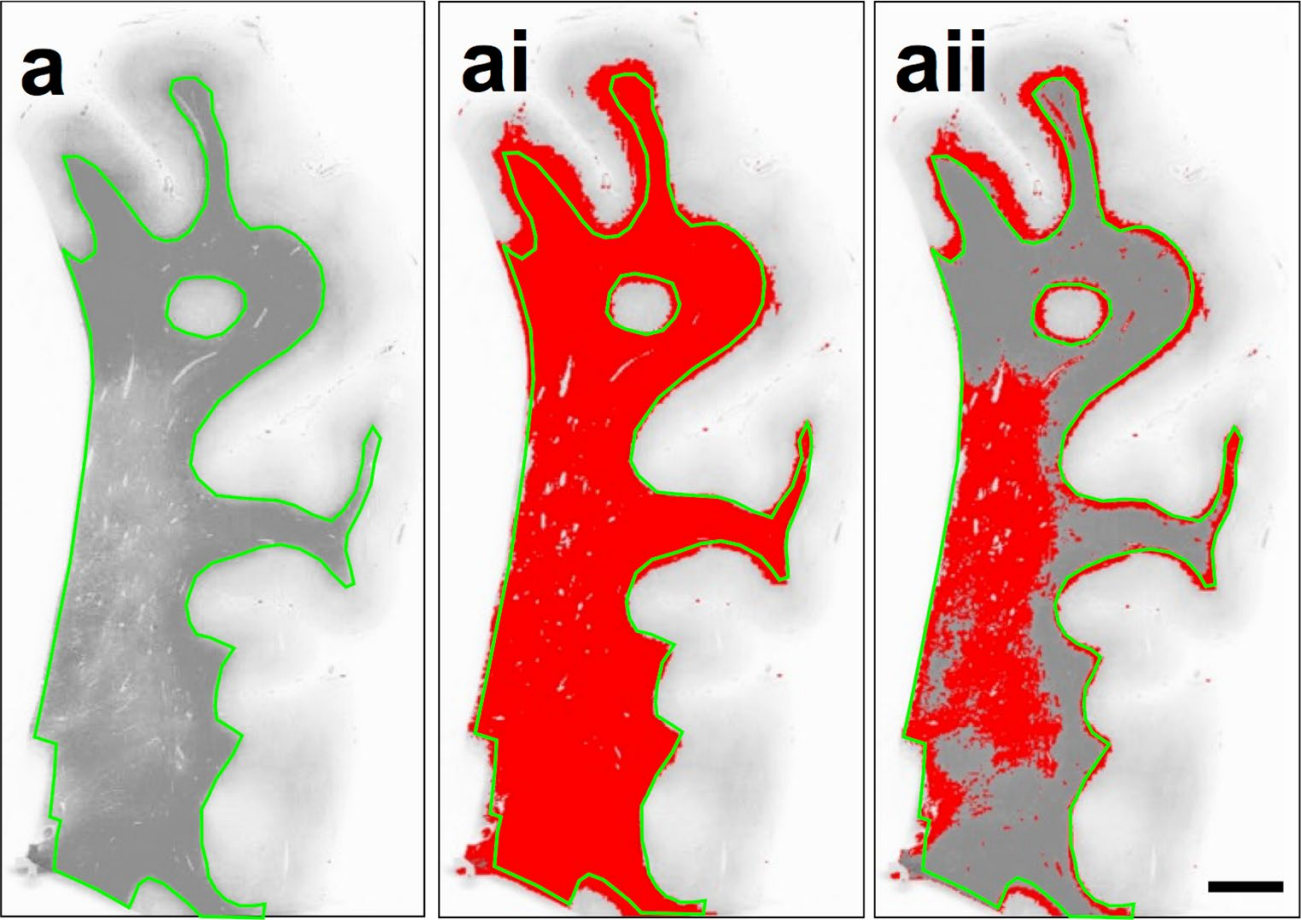
**a** LFB stained sections were scanned and monochrome images were uploaded into Image-Pro Plus software program where the white matter was manually delineated to create a region of interest (*green line*). **ai** Using manual adjustment of the 8-bit grey scale threshold, total white matter area measured within the region of interest only. **aii** Using manual adjustment of the 8-bit grey scale threshold the WML was measured. Percentage area of WML per total white matter area was calculated and is expressed in the manuscript as WML area (WMLA). *Scale bar* 1 cm, valid for **a**–**aii**

All image analysis was performed blinded to neuropathological diagnosis. Whole LFB stained sections were scanned using an Epson Perfection V700 scanner (dual lens system) and monochrome images were uploaded into Image-Pro Plus software program (Media Cybernetics Inc, USA; version 6.3). White matter area was manually delineated from the cortical ribbon using the freehand selection tool (Fig. 1a) and total white matter area recorded (Fig. 1ai). The WML area was identified based on a defined reduction in grey colour that is clearly differentiated from the surrounding normal appearing white matter (NAWM) with the naked eye, and was confirmed by the ‘bubbly’ like appearance and loosening of tissue under microscopic examination [54]. To differentiate between the WML and NAWM, the 8-bit grey scale threshold was manually adjusted to select only the WML area (Fig. 1aii). The percentage area of WML per total white matter area was calculated and expressed as percentage WML area (WMLA) to obtain a measure of overall WML severity. The integrated optical density (IOD) of the LFB stain was measured. The IOD is the amount of light able to transmit through a sample and represents a measure of the intensity of histological staining. In Image Pro Plus a pixel value of 0 represent black and 255 represents white, therefore, a higher value signifies a greater amount of light transmission, i.e. a lighter stain, which, regarding LFB staining, indicates a reduction of myelin. Three images from the WML and three images from the NAWM were randomly captured at 200× magnification using a Nikon 90i microscope and uploaded on Image-Pro Plus. The mean IOD per pixel was recorded for each image and a subsequent mean IOD per pixel for WML and NAWM per case were calculated.

**Fig. 2:**
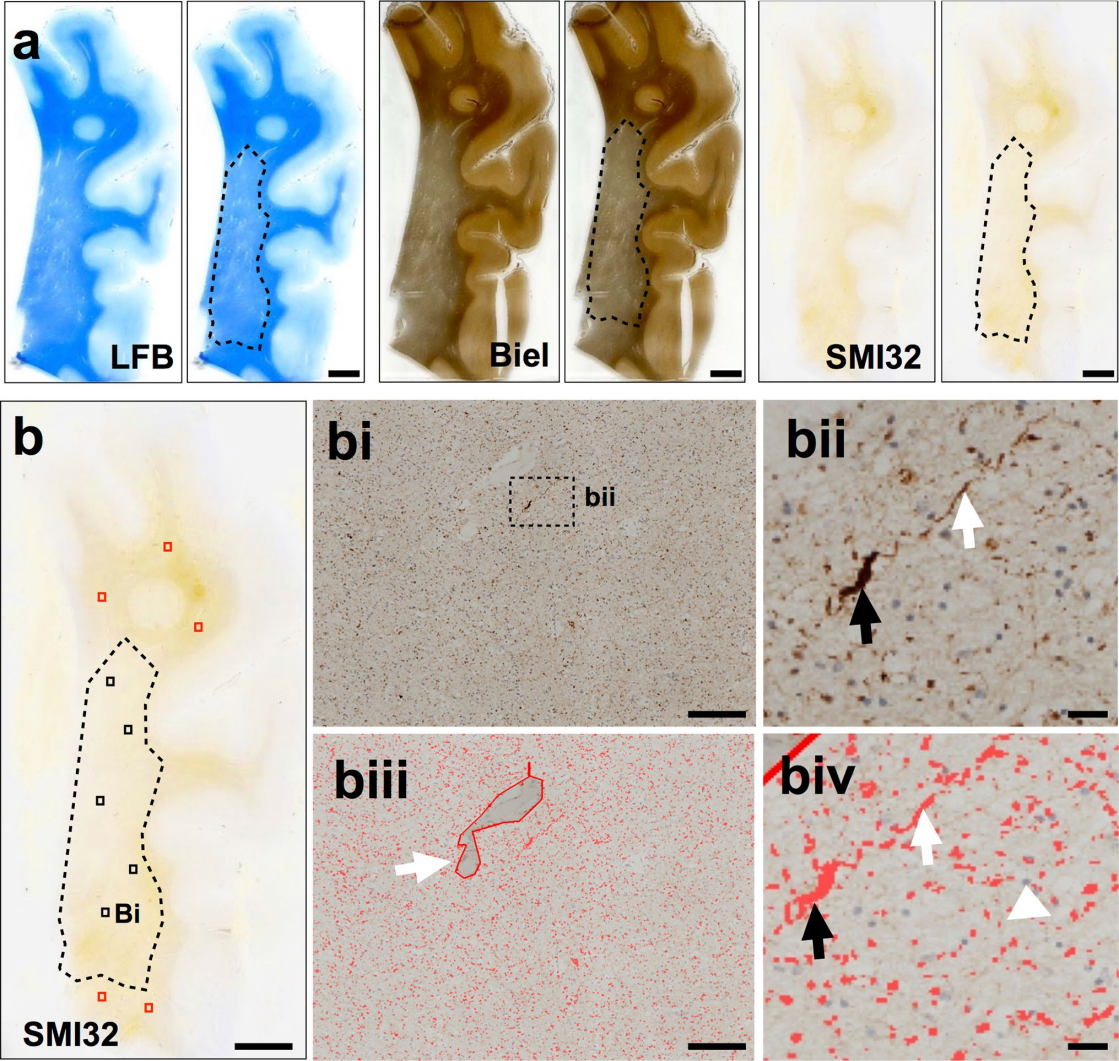
**a** WML were identified on LFB stained sections of parietal tissue and delineated from the NAWM by hand. This was used to identify the same WMLA on serial sections stained with Bielschowsky’s silver stain and SMI32. **b** A SMI32 stained section of parietal tissue indicating the five randomly selected areas for image capture in both the WMLA (*black boxes*) and NAWM (*red boxes*). **bi** 3 × 3 large image acquisition of SMI32-IR in WMLA. **bii** Magnified photomicroimage of SMI32 axonal positive pathology including discontinuous lines (*white arrow*) and end-bulbs axonal spheroid (*black arrow*). **biii** Image bi with applied bespoke SMI32 threshold including an example of artefact removal (*white arrow*). **biv** is image **bii** indicating discontinuous lines (*white arrow*) and end bull axonal spheroid (*black arrow*) with applied SMI32 threshold. Cellular nuclei were excluded from assessment (*white arrow head*). Mean area covered by IR was stated as a percentage of the total image area and the respective values are expressed as SMI32-IR. *LFB* luxol fast blue, *Biel* Bielschowsky’s silver stain, *WMLA* white matter lesion area, *NAWM* normal appearing white matter, *IR* immunoreactivity. *Scale bar* 50 mm, valid for all images in image **a** and image **b**; 100 μm, valid for **bi**, **biii**; 50 μm, valid for **bii**, **biv**

On LFB stained sections, the WMLA was identified macro- and microscopically and delineated by hand using a permanent marker pen to clearly differentiate between WML and NAWM. This delineated section was subsequently used to identify the corresponding WMLA in the adjacent Bielschowsky and SMI32 stained sections (Fig. 2a). On both Bielschowsky and SMI32 stained sections, 3 × 3 single images at 200× magnification were captured in WMLA and then combined to yield one large image representing an area of 1.7 mm^2^. This procedure was performed five times in each WML and NAWM to give a total of ten large images representing an area of 1.7 cm^2^ per section (Fig. 2b–bi). Using NIS elements version 3.0 (Nikon, Surrey, UK), individual and standardized Red Green Blue (RGB) thresholds for Bielschowsky’s staining and SMI32 immunoreactivity (IR) (axonal swellings and ovoids (Fig. 2bii)) were applied to the large images (Fig. 2biii–biv). If necessary, large images were subjected to manual setting of regions of interest to exclude vessels and perivascular spaces and artefacts (Fig. 2biii, white arrow). For the Bielschowsky’s staining, the RGB threshold was manually adjusted until all visible axons were included. For SMI32-IR, RGB intensity values for binary layer pixels were set as follows; R14-168, G4-158, B0-53. The area covered by Bielschowsky’s stain was measured and its percentage of the total measured area calculated and expressed as Bielschowsky’s area (BiA). As for Bielschowsky’s stain the percentage area covered by SMI32-IR was determined, but to adjust for overall axonal density SMI32-IR percentage areas were first divided by BiA and multiplied by 100 and the resulting values are expressed as SMI32-IR. For both BiA and SMI32-IR mean regional values were calculated for the WMLA and NAWM per case.

**Fig. 3:**
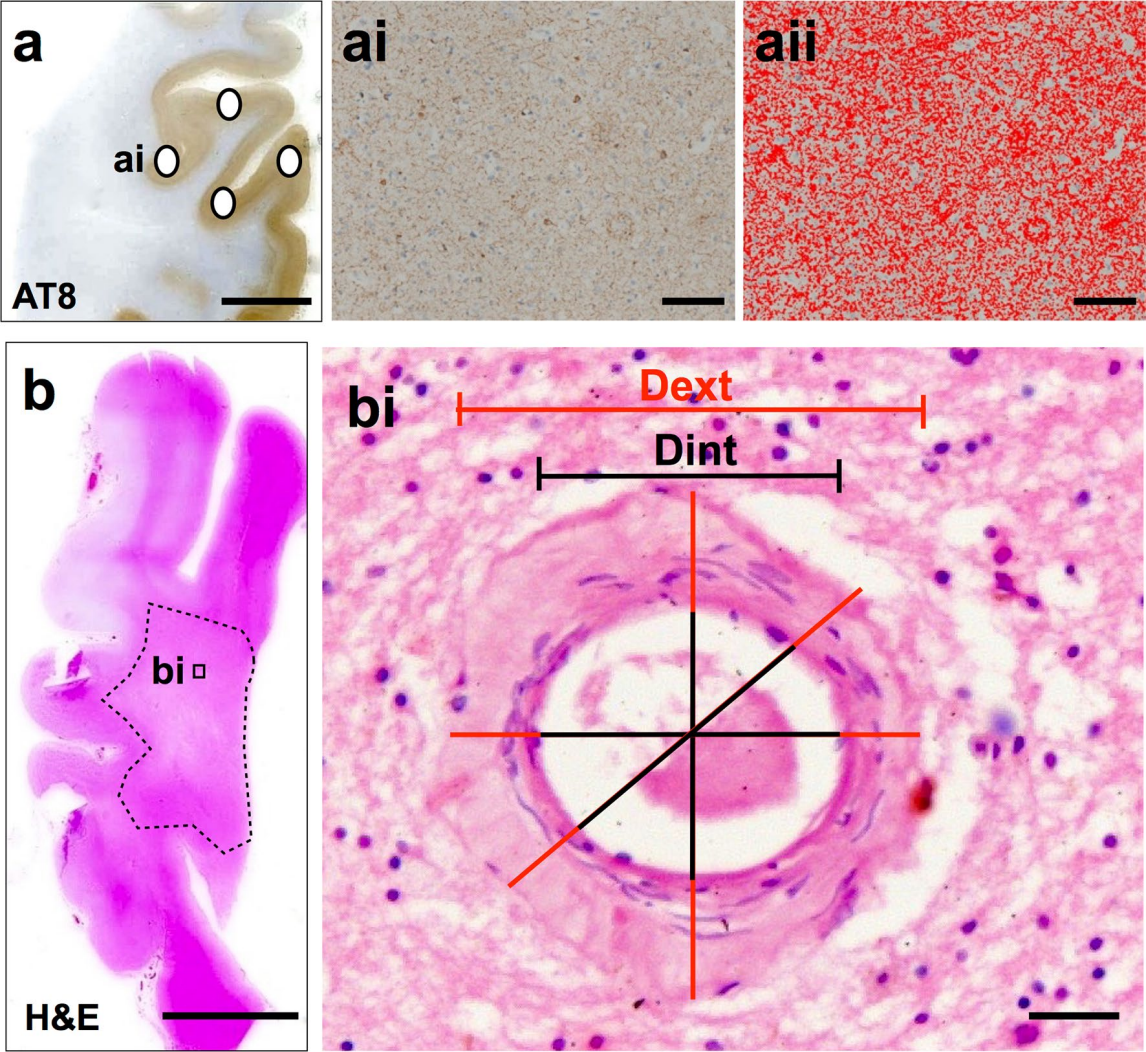
**a** AT8 stained parietal section indicating the four sample areas. **ai** 3 × 3 large image acquisition of cortex with AT8-IR. **aii** Image **ai** with applied bespoke threshold for AT8-IR only. Mean area covered by IR was stated as a percentage of the total image area and the respective values are expressed as AT8-IR. **b** H&E stained section of parietal tissue with deep white matter indicated (*dashed line*). Microphotoimages of up to eight individual white matter vessels were captured from the white matter for SI assessment. **bi** A microphotoimage of a white matter arteriole. SI was calculated using VasCalc software; [66] the internal and external diameters were measured three times to yield a SI value a mean SI value was calculated per section. *IR* immunoreactivity, *SI* sclerotic index, *Dext* external diameter, *Dint* internal diameter. *Scale bar* 1 cm, valid for images in **a**, **b**; 100 μm, valid for **ai**, **aii**; 50 μm, valid for **bi**

For the quantification of cortical protein aggregates, four areas of parietal cortical tissue were selected on AT8 and 4G8 immunostained tissue sections. The four parietal cortex sample areas included sulci, midsection and gyri to yield an accurate mean measure of cortical pathological burden as protein aggregates have been shown to be highest in the sulcus and lowest in the gyral tip [20] (Fig. 3a–ai). At each cortical location, 3 × 3 single images were captured at 200× magnification and combined into one large image as previously described. If necessary large images were subjected to manual setting of regions of interest to exclude white matter and meningeal structures. Standardized RGB thresholds were applied separately for AT8 and 4G8 (Fig. 3aii). RGB intensity values for binary layer pixels were set as follows; AT8: R25-170, G27-156, B11-126; 4G8: R50-180, G20-168, B8-139. In addition, we set a size restriction threshold for the assessment of 4G8, which excluded the measurement of immunoreactive signals with an area below 100 μm^2^; this was necessary to ensure that physiological APP that is stained with 4G8 antibody was not included in the measurement. The percentage areas covered by AT8 and 4G8 immunoreactivity were measured and the mean values for the four sample areas were calculated and are expressed as AT8-IR and 4G8-IR, respectively.

Sclerotic index (SI) is a surrogate marker of SVD severity. SI measurements were performed on H&E stained parietal tissue sections. SI was calculated using the formula SI = 1 − (internal diameter/external diameter); the SI of normal arteries and arterioles ranges from 0.2 to 0.3, while an SI of 0.3 to 0.5 indicates mild to moderate SVD and SI values >0.5 are seen in severe SVD [33]. A maximum of eight randomly selected cerebral white matter arteries and/or arterioles >50 μm diameters were identified from the whole white matter (Fig. 3b) and a single image captured at 200× magnification. SI was calculated on single vessel images using the software program VasCalc as previously described [66] (Fig. 3bi). Individual SI values were used to calculate a mean SI value for the parietal white matter in each case.

### Biochemistry

Frozen (−80 °C) coronal slices incorporating the parietal lobe were brought to −20 °C in a freezing cabinet and sub-dissected to remove Brodmann area 40/22 and its adjoining white matter. 10 μm frozen sections were cut and fixed in 10% buffered aqueous formaldehyde solution for 1 h and then histochemically stained with LFB as described above. On the slide, the WML was identified microscopically and delineated by hand to clearly differentiate between WML and NAWM and used to guide frozen tissue extraction. Approximately 250 mg of tissue was removed from each WML and the NAWM. Lysis buffer containing 0.2 M tetraethyl ammonium bicarbonate, pH 7.2 (TEAB; Sigma, UK) and protease inhibitor tablets (1 tablet per 10 ml; Complete, Roche, Burgess Hill, UK) were added to tissue samples at a 1 mg/1 ml ratio. Samples were then homogenised using an Ultra-turrax T10 homogeniser (S10N-5G 5 mm diameter probe; 30,000 rpm), aliquoted at 500 μl with 5 μl of 1% SDS and stored at −80 °C until required.

Total protein concentration was determined by Bradford assay and final protein concentration for all samples was adjusted to 3 mg/ml. Using commercially available sandwich enzyme-linked immunosorbent assays (ELISA) kits, five proteins were measured in both WML and NAWM tissue samples, including Wallerian degeneration markers calpain2 (CAPN2; large catalytic subunit (80 kDa) found in myelinated axons), calpain specific inhibitor calpastatin (CASP) [21], calpain specific spectrin breakdown product 145 (SBDP145) [68], and ischemia markers myelin-associated glycoprotein (MAG; myelin sheath protein, expressed only in the myelin loops and highly vulnerable under ischemic conditions) and proteolipid protein (PLP; myelin sheath protein that is abundant throughout the sheath and stable under ischemic conditions) [6] to calculate the MAG:PLP as an indication of pre-mortem white matter ischemia [6]. Details regarding ELISA kits are presented in Table 3. ELISA protocols were followed according to manufactures instructions. Briefly, working standards and samples were prepared/diluted as required. 100 μl of standard and sample were pipetted into wells of the 96-well coated microplates and incubated for 2 h at 37 °C. Liquid was removed and 100 μl of biotin-antibody was applied to each well and incubated for 1 h at 37 °C. Wells were then aspirated and washed three times with wash buffer before 100 μl HRP-avidin solution was applied to each well and incubated for 1 h at 37 °C. Wells were aspirated and washed a further five times and 90 μl of TMB substrate was applied to each well and incubated for 30 min at 37 °C. The reaction was stopped with 50 μl of stop solution per well and absorbance was read at appropriate wavelength within 5 min in a FLUOstar Omega multi-detection microplate reader (BMG Labtech, Aylesbury, UK).

**Table 3:** Details of ELISA kits used for detection of proteins in frozen tissue samples

Protein	ELISA Kit		Sample dilution	Standard curve (ng/ml)	OD wavelength
	Cat #	Description			
CAPN2	CSB-E17822h	Human CAPN2 ELISA kit, Cusabio	Neat samples	2.5–0.039	450 and 540 nm
CAST	CSB-E17483 h	Human CAST ELISA kit, Cusabio	1:50	100–1.56	450 and 540 nm
SBDP145	CSB-EQ028022HU	Human SBDP145 ELISA kit Cusabio	Neat samples	40–0.625	450 and 540 nm
MAG	CSB-E17901h	Human MAG ELISA Kit, Cusabio	Neat samples	20–0.1	450 and 540 nm
PLP	MBS266920	Human PLP ELISA Kit, MyBioSource	Neat samples	10–0.156	450 nm

### Statistics

The Statistical Package for Social Sciences software (SPSS ver. 21) was used for statistical evaluation. Variables were tested for normality using the Shapiro–Wilk test and visual inspection of variable histograms. Differences in related variables (WML vs. NAWM) were assessed using non-parametric Wilcoxon test. Group effects were assessed using either non-parametric (Mann–Whitney *U*) or parametric (independent samples *t* test) procedures. Where appropriate, Spearman’s (*ρ*) and Pearson’s (*r*) correlation coefficients were used to assess associations between variables. Exploratory stepwise linear regression analyses were also conducted to investigate pathological predictors of WMLA and WML pathology.

## Results

No significant differences were observed in age or post-mortem delay between AD and control groups. AD cases had significantly higher levels of AD neuropathological change and significantly lower MMSE scores compared to controls (Table 1). Fifty cases contained at least one WML and all 55 cases underwent NAWM assessment.

### Histological characteristics of WML and NAWM

Mean values for WMLA, BiA, LFB-IOD and SMI32-IR for both WML and NAWM are presented in supplementary Table 1. Briefly, with regards to the whole cohort, when compared to NAWM tissue, WML tissue had significantly lower BiA (*p* < 0.0001), indicating axonal loss, significantly higher LFB-IOD values (*p* < 0.0001), indicating demyelination, as well as significantly higher SMI32-IR (*p* < 0.01), indicating increased chronic axonal transport dysfunction. WMLA was significantly greater in AD compared to controls (*p* < 0.05), but SI values did not differ between these groups.

### Associations between WML severity and axonal loss, demyelination and axonal transport

**Fig. 4:**
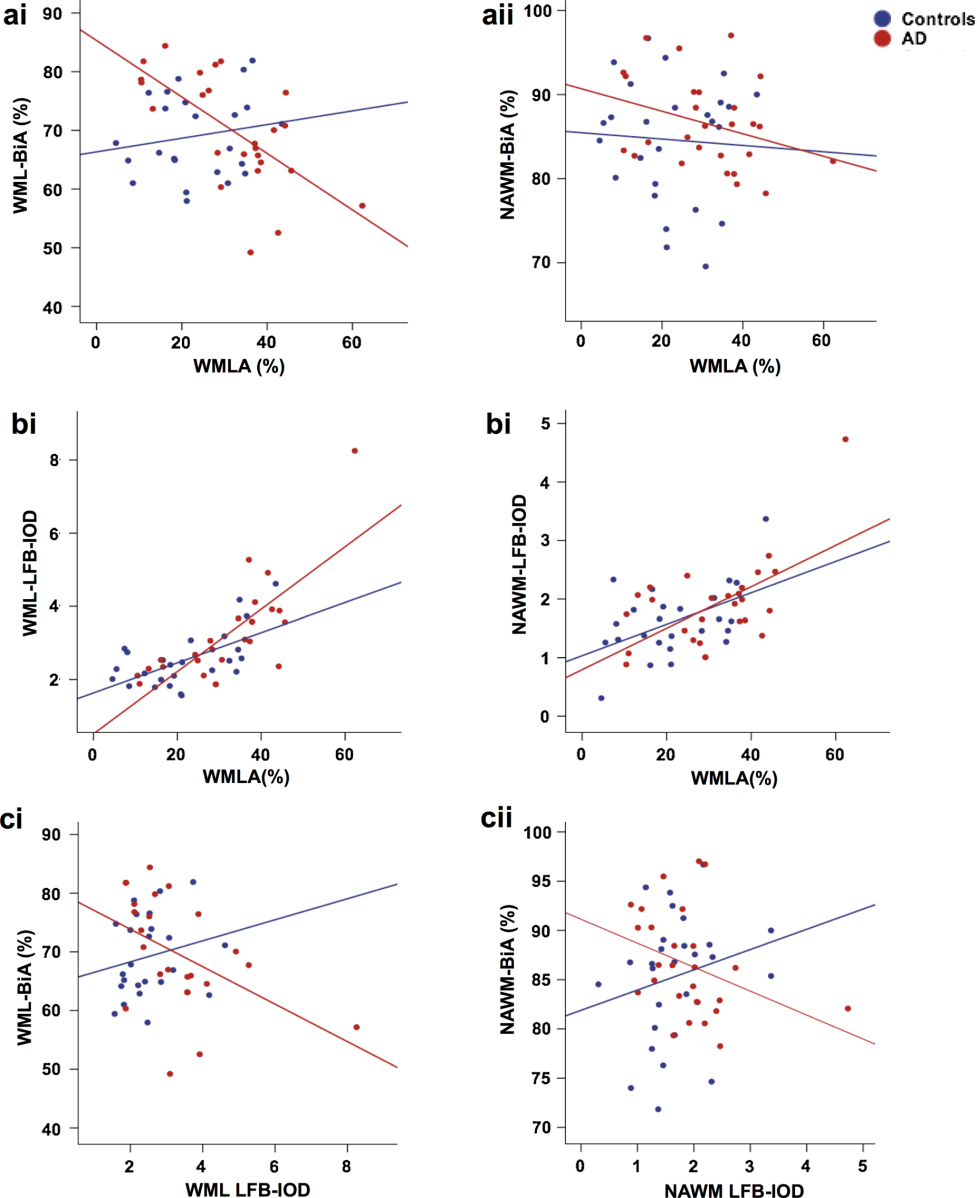
Scatter graphs show; **ai** negative correlations between WMLA and WML-BiA in AD cases only. **aii** Negative correlations between WMLA and NAWM-BiA in AD cases only. **bi** A positive correlation between WMLA and WML- and **bii** NAWM- LFB-IOD was seen in both AD and control cases. **ci** a negative correlation between WML-BiA and WML-LFB-IOD in AD cases only. **cii** A negative correlation between NAWM-BiA and NAWM-LFB-IOD was seen in AD cases only. *p* values and associated correlation coefficients are shown in main text. *WML* white matter lesion, *NAWM* normal appearing white matter, *WMLA* white matter lesion area, *BiA* Bielschowsky’s area, *LFB-IOD* luxol fast blue integrated optical density

We examined the relationship between WMLA and BiA, LFB-IOD, and SMI32-IR of both WML and NAWM. In AD cases, WMLA negatively correlated with both WML-BiA (*ρ* = −0.648, *p* = 0.0001; Fig. 4ai) and NAWM-BiA (*ρ* = −0.334, *p* = 0.044; Fig. 4aii). In contrast, no correlations were observed in control cases (WML-BiA, *p* = 0.436; NAWM-BiA, *p* = 0.365; Fig. 4ai–ii), indicating that increasing WML severity was significantly associated with axonal loss in the WML and surrounding NAWM in AD cases but not in controls. In both AD and controls, WMLA correlated with both WML-LFB-IOD (AD, *ρ* = 0.755, *p* = 0.0001; controls, *ρ* = 0.413, *p* = 0.016; Fig. 4bi) and NAWM-LFB-IOD (AD, *ρ* = 0.445, *p* = 0.01; *ρ* = 0.407, *p* = 0.018; Fig. 4bii), indicating increasing WML severity was associated with demyelination in the WML and surrounding NAWM in both AD and control cases. Negative correlations were observed between BiA and LFB-IOD in both WML (*ρ* = −0.528, *p* = 0.004) and NAWM (*ρ* = −0.430, *p* = 0.013) in AD cases only (controls: WML, *p* = 0.133; NAWM, *p* = 0.073) (Fig. 4ci–ii). This indicates that in AD cases loss of myelin is primarily due to loss of myelinated axons, unlike control cases that exhibited no such association. No correlations were revealed between WML-SMI32-IR or NAWM-SMI32-IR and WMLA in either AD (WML, *p* = 0.390; NAWM, *p* = 0.186) or control cases (WML, *p* = 0.091; NAWM, *p* = 0.402). To investigate whether WML-BiA or WML-LFB-IOD independently predicted WMLA, stepwise linear regression analysis was performed. In the AD group both WML-BiA (*β* = −0.375, *p* = 0.013) and WML-LFB-IOD (*β* = 0.761, *p* = 0.0001) significantly predicted WMLA (model *R* ^2^ = 0.688, *F*_(2)_ = 23.18, *p* < 0.001). In contrast for controls, only WML-LFB-IOD was found to be a significant predictor of WMLA (model *R* ^2^ = 0.332, *F*_(2)_ = 12.41, *p* < 0.01; *β* = 0.601, *p* = 0.002) and not WML-BiA (*p* = 0.957).

### Influence of cortical neurodegenerative pathology, SVD and CAA on WML severity, axonal loss, demyelination and axonal transport

We investigated whether cortical neurodegenerative AD-pathologies and SVD were associated with white matter pathological changes. A correlation matrix for the whole cohort, as well as separately for AD and controls, is presented in supplementary Table 2. No correlations were seen when statistical analysis was performed separately for AD and controls. However, with regards to the whole cohort, which is inclusive of Braak stage 0–VI and Thal Aβ Phases 1–5, WMLA correlated with AT8-IR (*ρ* = 0.284, *p* = 0.022) but not 4G8-IR (*p* = 0.072), SI (*p* = 0.451) or CAA (*p* = 0.185). WML-LFB-IOD was also correlated with AT8-IR (*ρ* = 0.289, *p* = 0.002) but not 4G8-IR (*p* = 0.147), SVD (*p* = 0.433) or CAA (*p* = 0.492). No correlations were observed between WML-BiA and any neurodegenerative pathology, SI or CAA. Finally, we correlated WML-SMI32-IR with AT8-IR, 4G8-IR, SI and CAA to determine if AD-associated pathologies or SVD was involved in chronic axonal transport dysfunction; however, no significant correlations were yielded with any pathology (AT8, *p* = 0.274; 4G8, *p* = 0.153; SI, *p* = 0.314; CAA, *p* = 0.916).

### Wallerian degeneration and ischemia

Firstly, we investigated the regulation and activation of the protease calpain2; calpain2 was increased in the WML compared to NAWM (*p* = 0.073) and calpastatin (calpain2 inhibitor) levels were decreased in the WML compared to NAWM (*p* = 0.10) indicating corresponding down regulation of calpastatin and up regulation of calpain2. A correlation between mean values of calpain2 and SBDP145 (*ρ* = 0.521, *p* = 0.0001) was observed, indicating specific calpain2 activity.

**Fig. 5:**
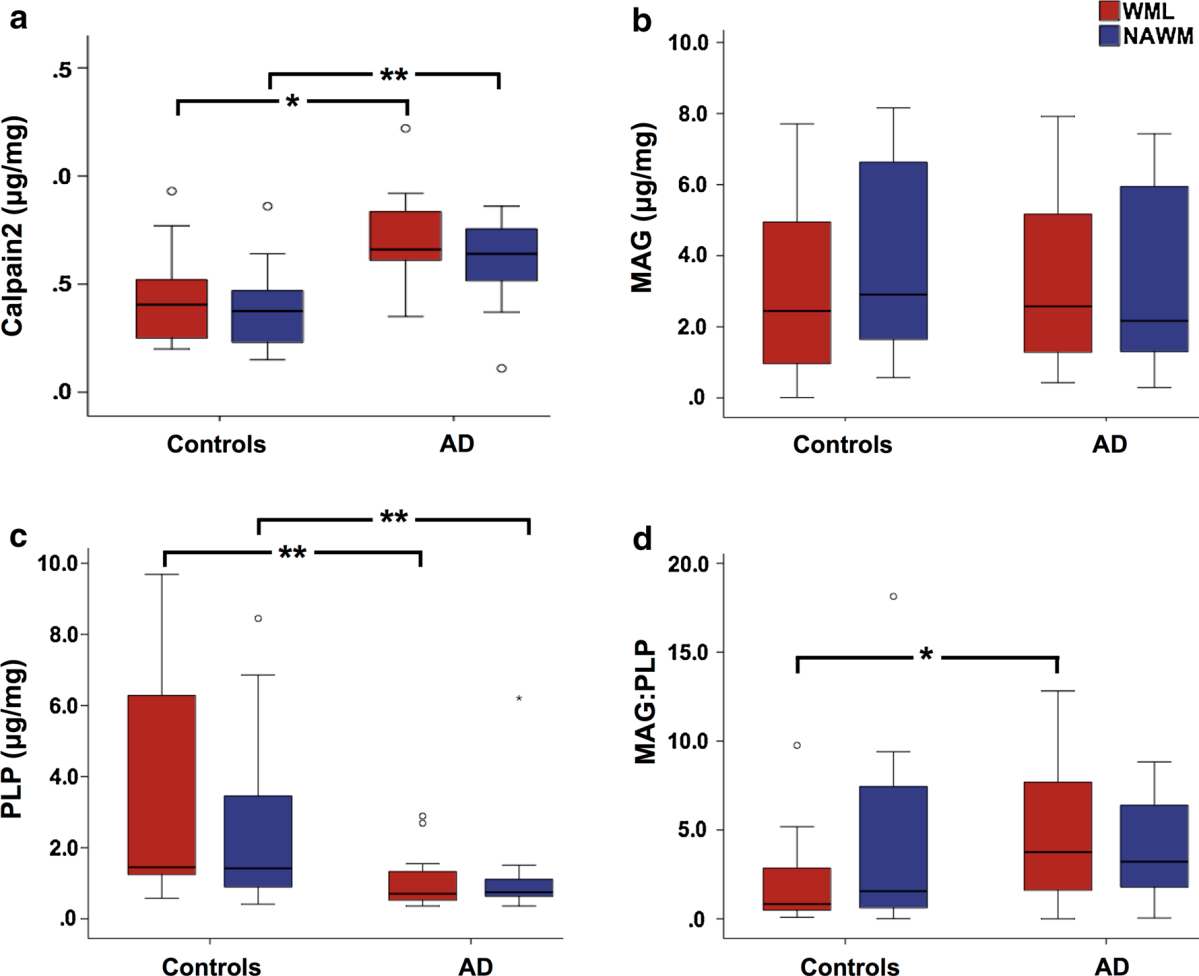
*Box plots* show; **a** Calpain2 was significantly higher in the WML tissue of AD cases compared to controls. **b** No significant difference was seen in WML-MAG between AD and control cases. **c** PLP was significantly lower in the WML tissue of AD cases compared to controls. **d** MAG:PLP was significantly lower in the WML tissue of control compared to AD cases. *p < 0.05; ***p < 0.001. Exact *p* values are shown in main text. *AD* Alzheimer’s disease, *MAG* myelin associated glycoprotein, *PLP* proteolipid protein

Calpain2 was significantly higher in both the WML (*p* = 0.022) and NAWM tissue (*p* = 0.005) of AD cases compared to controls (Fig. 5a). No significant difference was revealed with WML- or NAWM MAG values between AD and control cases (WML, *p* = 0.457; NAWM, *p* = 0.460; Fig. 5b), in contrast to WML- and NAWM PLP that was found to be significantly reduced in AD cases (WML, *p* = 0.0001; NAWM, *p* = 0.007; Fig. 5c). The MAG:PLP was significantly lower in WML tissue of control cases compared to AD cases (*p* = 0.02; Fig. 5d), however, no significant differences was seen in NAWM tissue between AD and controls (*p* = 0.221).

With regards to the whole cohort that is inclusive of Braak NFT stage 0–VI and Thal Aβ Phases 1–5, we examined correlations of WML measures of calpain2, MAG, PLP and MAG:PLP with pathological measures of cortical neurodegenerative AD-pathologies, SVD and CAA to investigate the pathological relationships of white matter changes. Calpain2 correlated with AT8-IR (*ρ* = 0.369, *p* = 0.01) and 4G8-IR (*p* = 0.012), but not SI (*p* = 0.184) or CAA (*p* = 0.433). No correlations were seen between MAG values and AT8-IR (*p* = 0.163), 4G8-IR (*p* = 0.256), SI (*p* = 0.210) or CAA (*p* = 0.590). PLP negatively correlated with both AT8-IR (*ρ* = −0.488, *p* = 0.001) and 4G8-IR (*ρ* = −0.401, *p* = 0.004), but not with SI (*p* = 0.273) or CAA (*p* = 0.536). MAG:PLP correlated with both AT8-IR (*ρ* = 0.347, *p* = 0.028) but not 4G8-IR (*p* = 0.076), SI (*p* = 0.705) or CAA (*p* = 0.453). No correlations were seen when statistical analysis was performed separately for AD and controls.

## Discussion

Here, for the first time, we reveal differences in the underlying pathology and molecular signatures of parietal WML between AD and non-demented elderly that indicate differing aetiologies. Pathologically, increasing severity of WML in AD is associated with both axonal loss and demyelination, in contrast to non-demented individuals in which WML are associated with demyelination only. Biochemical analysis indicated an increase in calpain2 levels in the white matter of AD cases, which is suggestive of increased Wallerian degeneration that is associated with increasing cortical burden of AD-related pathologies but not with SVD. Furthermore, MAG:PLP values were reduced in the WML tissue of control cases, indicating hypoperfusion. Overall, our data indicate that parietal WML (WMH) seen in AD are associated with demyelination and axonal loss as a result of degenerative axonal loss and not ischemic damage as seen in normally aged controls.

The pathogenesis of WML is often being assumed to be the result of SVD; hence, the detection of in vivo WMH frequently supports the diagnosis of VCI/VaD. However, ample evidence has indicated that WML in AD may well be the result of degenerative axonal loss due to Wallerian degeneration triggered by cortical AD pathology. Indeed, we found that WML were significantly more severe in the AD cases compared to the non-demented cases, in agreement with findings in previous studies [8, 40, 49]. Our thorough, quantitative neuropathological investigations revealed that in AD axonal loss and demyelination, in both the WML itself and the surrounding NAWM, were associated with and are a predictor of increasing severity of WML. This is in contrast to non-demented cases where only demyelination predicts and associates with WML severity. These pathological changes were further supported by our biochemical analysis of WML tissue, which showed a significant decrease in PLP levels in AD, indicative of  axonal atrophy, as well as equal loss of MAG levels between AD and control brains, signifying demyelination in both groups. Both the pathological and biochemical data indicate that increased myelin loss in the AD group is likely due to severe axonal atrophy. A previous neuropathological study investigated differences in myelin and axonal densities of WML between AD and controls cases and found no differences; however, this was based on WML from any brain region and was not specific to the posterior or medial temporal regions [23]. We can, therefore, report for the first time differences in the pathological composition WML between AD cases and controls in the parietal white matter.

Degenerative axonal loss in AD can occur as a consequence of Wallerian degeneration [15, 29, 65] where calpain is the primary protease involved in axonal destruction [38]. Our data revealed that calpain levels were significantly increased in WML in AD compared to controls and were associated with increasing cortical HPτ and Aβ pathology burden but not with SVD. Calpain activation is thought to be triggered by anterograde axonal transport disruption and/or blockage [12, 39], which are a common early feature in AD. Axonal SMI32 pathology, which is an indicator of chronic axonal transport dysfunction [48], was found to be significantly higher in WML compared to NAWM indicating disruption of axonal transport in the WML area. However, no difference in the amount of SMI32 pathology was found between AD and controls, and therefore, we cannot conclude whether axonal transport dysfunction is the instigator of increased levels of calpain in the AD group.

WML in the control cases, that primarily consisted of demyelination, were found to be the result of ischemia as indicated by a significant reduction in the MAG:PLP ratio. Barker and colleagues had previously measured the MAG:PLP ratio in the parietal white matter of AD and control cases and found no respective differences. However, they did not distinguish specifically between NAWM and WML [6], hence the data from Barker and colleagues is in agreement with our findings in the NAWM. Only in the WML tissue of control cases was the MAG:PLP ratio significantly lower compared to WML seen in AD cases indicating that in AD WML were not primarily associated with hypoxia. Ischemia has long been associated as the primary cause of WML and WMH in both normal ageing and dementia [24, 45, 47]; however, with the addition of more sensitive and reliable biochemical measures of ischemic change [6] we could now clarify that ischemia is not the only potential cause for WML and it appears unlikely to be the major cause for posterior WML in AD. Naturally, SVD in AD cases may have an influence on the development of WML; however, our data indicates that the respective influence of HPτ pathology exceeds that of SVD.

Cortical HPτ pathology was associated with both WML severity and demyelination, while the latter were not associated with arteriopathy in cases showing considerable cortical HPτ. While no direct pathological associations between HPτ or Aβ pathology burden with decreased axonal density were revealed, a decrease in WML PLP levels (indicative of axonal atrophy) was associated with increasing HPτ pathology. One may speculate that the increasing burden of cortical HPτ pathology is a trigger of Wallerian degeneration. Indeed, cortical HPτ has been implicated as an instigator of degenerative axonal loss in AD. Previous quantitative neuropathological [40], longitudinal community-based [52] and biomarker studies [60] have indicated an association between HPτ and the development of WMH in the absence of SVD [17, 35, 60]. Moreover, in Pick’s disease, which is a primary tauopathy, white matter degeneration and demyelination was significantly more severe in white matter regions proximal to severe cortical HPτ pathology [58]. However, the exact underlying mechanisms of HPτ-associated axonal degeneration are still unclear and further mechanistic investigations are warranted.

It is important to note that our study focused specifically on WML located in the parietal region. Studies have indicated that in non-demented patients WML/WMH are more prevalent in the frontal region compared to patients with AD [61, 64] and are attributed to cerebrovascular disease [61, 62]. The difference in aetiology could be attributed to the posterior and MTL regions being affected early with HPτ pathology deposition [10]. Differentiation of the aetiology and pathological/biochemical signatures of WML/WMH from the anterior and posterior regions of the brain is warranted for diagnostic clarification.

CAA was not associated with any measure of WML pathology or molecular measure of Wallerian degeneration or ischemia in agreement with a previous neuropathological and imaging study [56]. Due to the high prevalence of CAA in AD statistical associations between CAA and WML may possibly be caused by the presence of concomitant cortical AD pathology, specifically HPτ pathology.

Understanding the underlying mechanisms of WML/WMH pathogenesis is crucial for the accurate diagnosis and therapeutic interventions of patients with cognitive impairment, in particular, as current clinical criteria for VaD/VCI [22] and mixed AD/VaD [16] are based on the detection of WMH as surrogate marker for SVD. Our findings provide further evidence that the underlying aetiology of WML in AD is not restricted to SVD and may indeed be indicative for AD pathology. If WML are invariably used as a surrogate biomarker for SVD, as suggested by current clinical guidelines [16, 22], this will bias cohort stratification in clinical trials, result in misdiagnosis of patients, and have a detrimental impact on the choice of therapeutic interventions, e.g. AD patients being misdiagnosed with VaD would not receive acetylcholinesterase inhibitors as recommended for managing mild to moderate AD [18]. As mentioned previously, our study focused on parietal WML and prior work suggests that the aetiology of WML differs by anatomical location [34], hence, further imaging studies that also involve new in vivo imaging of HPτ [11] and Aβ [32] pathology and integrate anatomical location, pathophysiology and impact on cognition are warranted. Finally, our results call for more detailed neuropathological evaluation of cerebral white matter injury in dementia. Current approaches do not adequately assess this important tissue and understanding the pathophysiology of white matter damage is not only important to clinical phenotype, but may open avenues to new therapeutics to modify the course of degenerative dementias.

In conclusion, the pathological and biochemical signatures of parietal WML/WMH differ between AD and non-demented individuals, and in AD the pathogenesis of posterior WML/WMH is associated with degenerative axonal changes probably occurring secondary to cortical AD-pathology. These novel findings support previous neuroimaging studies that indicate Wallerian degeneration-associated axonal damage may be a cause of posterior WMH in AD. Further elucidation of the role of AD pathologies in the pathogenesis of WML/WMH is paramount for accurate diagnosis and improved stratification of patients for clinical trials.

## Electronic supplementary material

Below is the link to the electronic supplementary material.

Supplementary material 1 (DOCX 88 kb)

Supplementary material 2 (DOCX 101 kb)
