# Supplementary material for: Parietal white matter lesions in Alzheimer’s disease are associated with cortical neurodegenerative pathology, but not with small vessel disease
Source: Acta Neuropathol. 2017 Jun 21;134(3):459–73. doi: 10.1007/s00401-017-1738-2 (PMC5563333; doi:10.1007/s00401-017-1738-2)
Supplement: Supplementary file 1 — Supplementary material 1 (DOCX 88 kb) [file 401_2017_1738_MOESM1_ESM.docx]

Supplementary table 1: Quantitative neuropathological measurements

|  | **Whole cohort**  (n = 55) | **WML vs. NAWM Statistic**  **(Wilcoxon test)** | **AD** (n = 27) | **Control** (n = 28) | **AD vs. control**  **(Statistic*_(df)_, p*-value)** |
| --- | --- | --- | --- | --- | --- |
| WMLA (%) | 26.71 (± 12.68) | - | 31.05 (± 12.68) | 22.2 (± 11.04) | U_(48)_ = 208, p = 0.011 |
| WML-BiA (%) | 69.48 (± 8.23) |  | 70.09 (± 9.51) | 68.87 (± 6.89) | U_(48)_ = 272, p = 0.432 |
| NAWM-BiA (%) | 85.63 (± 6.23) | p = 0.00001 | 86.53 (± 5.3) | 84.76 (± 7.00) | U_(53)_ = 352, p = 0.662 |
| WML-LFB-IOD | 2.89 (± 1.17) |  | 3.23 (± 1.37) | 2.58 (± 0.85) | U_(48)_ = 230, p = 0.031 |
| NAWM-LFB-IOD | 1.78 (± 0.72) | p = 0.00001 | 1.89 (± 0.75) | 1.68 (± 0.67) | U_(53)_ = 293, p = 0.216 |
| WML-SMI32-IR | 0.28 (± 0.93) |  | 0.085 (± 0.20) | 0.083 (± 0.19) | U_(48)_ = 218, p = 0.990 |
| NAWM-SMI32-IR | 0.04 (± 0.08) | p = 0.002 | 0.044 (± 0.98) | 0.040 (± 0.80) | U_(53)_ = 319, p = 0.321 |
| Cortical AT8-IR | - | - | 17.11 (± 12.75) | 0.25 (± 0.68) | U_(53)_ = 7.5, p = 0.000 |
| Cortical 4G8-IR | - | - | 9.8 (± 6.91) | 1.5 (± 2.92) | U_(53)_ = 63, p = 0.000 |
| WM SI value | - | - | 0.37 (± 0.067) | 0.36 (± 0.089) | t_(53)_ = -754, p = 0.454 |

Abbreviations: AD, Alzheimer’s disease; *df*, degrees of freedom; WML, white matter lesion; NAWM, normal appearing white matter; BiA, Bielschowsky’s area - represents axonal density; LFB-IOD, luxol fast blue integrated optical density- representing myelin pallor; SMI32-IR, SMI32 immunoreactivity- represents SMI32 pathology burden; t, Independent samples test; U, Mann-Whitney U test; AT8, hyperphosphorylated tau pathology, 4G8, amyloid-beta pathology; WM, white matter; SI, sclerotic Index
