# Supplementary material for: Parietal white matter lesions in Alzheimer’s disease are associated with cortical neurodegenerative pathology, but not with small vessel disease
Source: Acta Neuropathol. 2017 Jun 21;134(3):459–73. doi: 10.1007/s00401-017-1738-2 (PMC5563333; doi:10.1007/s00401-017-1738-2)
Supplement: Supplementary file 2 — Supplementary material 2 (DOCX 101 kb) [file 401_2017_1738_MOESM2_ESM.docx]

Supplementary table 2: Correlation matrix of neuropathological variables for whole cohort and AD and controls

| **Whole cohort** | **WML area (%)** | **WML-Bielschowsky's** | **WML-LFB OD** | **WML-SMI32-IR** |
| --- | --- | --- | --- | --- |
| **Cortical AT8-IR** | ρ = 0.284, p = 0.022 | ρ = 0.158, p = 0.139 | ρ = 0.289, p = 0.02 | ρ = 0.098, p = 0.274 |
| **Cortical 4G8-IR** | ρ = 0.203, p = 0.072 | ρ = 0.056, p = 0.350 | ρ = 0.147, p = 0.147 | ρ = -0.162, p = 0.153 |
| **WM SI** | r = 0.018, p = 0.451 | r =-0.162, p = 0.134 | r = 0.024, p = 0.433 | r = 0.078, p = 0.314 |
| **CAA** | ρ = 0.187, p = 0.185 | ρ = 0.05, p = 0.734 | ρ = 0.097, p = 0.492 | ρ = 0.017, p = 0.916 |
| **AD** |  |  |  |  |
| **Cortical AT8-IR** | ρ = -0.127, p = 0.264 | ρ = 0.079, p = 0.353 | ρ = -0.166, p = 0.209 | ρ = 0.319, p = 0.069 |
| **Cortical 4G8-IR** | ρ = 0.125, p = 0.267 | ρ = 0.045, p = 0.416 | ρ =- 0.274, p = 0.088 | ρ =- 0.319, p = 0.069 |
| **WM SI** | r = -0.029, p = 0.444 | r =-0.192, p = 0.185 | r = -0.148, p = 0.240 | r = 0.123, p = 0.293 |
| **CAA** | ρ = 1.00, p = 0.628 | ρ = 0.055, p = 0.799 | ρ = 0.123, p = 0.560 | ρ = 0.038, p = 0.867 |
| **Controls** |  |  |  |  |
| **Cortical AT8-IR** | ρ = 0.103, p = 0.308 | ρ = 0.131, p = 0.272 | ρ = 0.344, p = 0.046 | ρ = 0.122, p = 0.314 |
| **Cortical 4G8-IR** | ρ = -0.288, p = 0.077 | ρ = -0.106, p = 0.307 | ρ = 0.142, p = 0.240 | ρ = -0.170, p = 0.243 |
| **WM SI** | r = 0.032, p = 0.440 | r =-0.152, p = 0.233 | r = 0.446, p = 0.238 | r = 0.009, p = 0.486 |
| **CAA** | ρ = 0.043, p = 0.834 | ρ = 0.022, p = 0.915 | ρ = 0.044, p = 0.829 | ρ = 0.138, p = 0.552 |

Abbreviations: WML, white matter lesion; LFB, luxol fast blue; IR, immunoreactivity; SI, sclerotic index; CAA, cerebral amyloid angiopathy; ρ, Spearman’s correlation coefficient; ρ', partial Spearman’s correlation coefficient controlled for AT8-IR; r, Pearson’s correlation coefficient; ρ', partial Pearson’s correlation coefficient controlled for AT8-IR.
